# A protocol of homozygous haploid callus induction from endosperm of *Taxus chinensis* Rehd. var. *mairei*

**DOI:** 10.1186/s40064-016-2320-4

**Published:** 2016-06-01

**Authors:** Yan-Lin Li, San-Wen Huang, Jia-Yin Zhang, Feng-Jiao Bu, Tao Lin, Zhong-Hua Zhang, Xing-Yao Xiong

**Affiliations:** Hunan Provincial Key Laboratory of Crop Germplasm Innovation and Utilization, Changsha, 410128 Hunan People’s Republic of China; College of Horticulture and Landscape, Hunan Agricultural University, Changsha, 410128 Hunan People’s Republic of China; The Institute of Vegetables and Flowers, Chinese Academy of Agriculture Sciences, Beijing, 100081 People’s Republic of China; State Key Laboratory of Subhealth Intervention Technology, Changsha, 410128 Hunan People’s Republic of China

**Keywords:** *Taxus*, Haploid, Endosperm, Callus initiation, Homozygote, SNP mark

## Abstract

Obtainment and characterization of the novel endosperm callus of *Taxus chinensis* Rehd. var. *mairei* are valuable for haploid breeding, genome, and functional genome in *Taxus*. Callus was obtained by hydropriming with sterile water for 3 days and suitable medium composition. The highest callus induction (70.89 %) and lower browning ratio (7.95 %) were obtained from Gamborg (B_5_) medium supplemented with 30 g l^−1^ of sucrose, 2.5 mg l^−1^ of 2,4-dichlorophenoxyacetic (2,4-D), 0.5 mg l^−1^ of 6-benzylademine (6-BA) and 7 g l^−1^ of agar under dark conditions. The auxin of 2,4-D had a better efficiency of callus induction than naphthylacetic acid, and over 1 mg l^−1^ of 6-BA was inhibitory to the callogensis of endosperm. The endosperm callus was haploid which was detectable by the flow cytometry. The genome block of homozygosity of callus was homozygous which was indicated by PCR-based SNP marks. The homozygous haploid of endosperm callus in vitro culture may be useful tools for taxoid-metabolism of gene engineering and bio-fermentation engineering.

## Background

*Taxus* plants are well-known by their ornamental values, cancer-inhibitory alkaloid paclitaxel (Taxol), and timber uses (Xi et al. 2014). As an effective curing cancer of chemical compounds for 50 years (Foa et al. 1994; Bi et al. 2014), the same difficulties with other supply of active compounds: limited quantity of active compounds in tissues, low growth rate, limited localization of active ingredients in the specific organs, and destruction of the nature resource (Kazmi et al. 1991; Frense 2007; Hussain et al. 2013). Additionally, chemical synthesis, semi-synthesis, heterologous expression systems, and plant cell culture are the main available resources for harvesting of taxoids, especially paclitaxel (Howat et al. 2014). However, the structure complexity, incomplete biosynthetic pathway and exogenous genes that lead to growth retardation due to taxadiene toxic, which caused producing low yields and numerous toxic side products by chemical synthesis, sim-synthesis, and heterologous expression systems (Howat et al. 2014; Li et al. 2014). The plant cell culture is seen as a successful approach of production paclitaxel without geographical and seasonal variations, and providing paclitaxel with continuous and uniform quality (Howat et al. 2014), while this issued with genetic instability, heterogeneous culture, low growth rates compared to bacterial (Kolewe et al. 2008). Bridging the sim-synthesis including of heterologous expression systems and plant cell culture will be allowed large-scale access to taxol compound, and analyzing the paclitaxel metabolisms pathway remain an important goal (Howat et al. 2014). Artificial cultivation of haploid materials (including of callus and haploids), which have been useful in research areas as mutation studies, gene mapping and genomics and as targets for transformation, may accelerate the realization of this goal.

Haploids are sporophytes of higher plants with gametophytic chromosome number (n instead of 2n), and it rarely occurs spontaneously which can be induced through several methods, such as in vivo pollination methods (wide hybridization, chromosome elimination, pollination with irradiated pollen and so on) and immature gametophytes (Germanà 2011). Endosperm as a storage tissue for nutrients which is a unique feature of flowering plants in angiosperms, and it is formed during a double fertilization event. Therefore, the embryo is diploid (2C) and the endosperm is triploid (3C) (Wang et al. 2009; Thomas and Chaturvedi 2008). Other seed plants (gymnosperms) of endosperm, as a nutritive tissue supporting the embryo growth, arises from the haploid (1C) cells of the female gametophyte (Wang et al. 2009). In this study, we investigated the effects of hydropriming, media compositions, auxins and cytokinins on endosperm callus induction of *Taxus chinensis* Rehd. var. *mairei*, and fully dissect the ploidy level and the genomic homozygosity of them.

## Methods

### Plant material

Fruits and twigs with needles of *T. chinensis* Rehd. var. *mairei* were collected from natural habitats in China in December, 2012. Seeds were stored at 4 °C after natural drying. Twigs with leaves, which were kept at 4 °C for no more than 7 days, were used as materials for FMC, DNA extraction and PCR amplification analysis. After the outer seed coat was removed, the seed with inner seed coat was surface-sterilized in 70 % (v/v) ethanol for 30 s, followed by a soaking in 0.1 % HgCl_2_ for 10 min. Subsequently, they were rinsed for several times in sterile water to completely remove the sterilizing agent.

### Basic medium

Six culture media were tested as follows: BLG, B_5_, MS, WPM, SH and 1/2MS. All six basic media contained 2.5 mg l^−1^ of 2,4-D, and 0.5 mg l^−1^ of 6-BA. Culture medium was solidified with 0.7 % agar and supplemented with 3 % (w/v) sucrose. After sterile seed with inner coat was immersed in sterile water for 1 day, endosperm was aseptically excised from embryo using forceps and a scalpel, and then it was transferred onto the culture medium. The highest callus ratio and lowest brown ration were considered as option index for estimation of optimal basic medium. All treatments were performed in triplicate, and each treatment consisted of 50 plantlets.

### Pre-treatment and illumination

Sterile seeds with inner coat were soaked in sterile water for 0, 1, 3, 5 and 7 days, respectively. They were used as endosperm donors for the callus induction. The optimal hydropriming time was selected. The efficiency of sterile water and sterile gibberellin III solution (3 mg l^−1^ of GA_3_) for 3 days on callus induction was compared. And effect of light condition with incubating in a growth chamber under a 16:8 h photoperiod at 112 μmol m^−2^ s^−1^ light provided by fluorescent lamps and darkness on callus induction was investigated. Endosperm was divided into two pieces by forceps and a scalpel without embryo, and then transferred onto B_5_ medium supplemented with 2.5 mg l^−1^ of 2,4-D and 0.5 mg l^−1^ of 6-BA. All treatments were performed in triplicate, and each treatment consisted of 50 plantlets.

### Effects of plant growth regulators

After soaking in sterile water for 3 days, endosperm was divided into two pieces by forceps and a scalpel without embryo. The B_5_ medium was supplemented with 0.5 mg l^−1^ of 6-BA and 1.0–4.5 mg l^−1^ of 2,4-D or naphthylacetic acid (1.0–4.0 mg l^−1^ of NAA), respectively (Table 2). B_5_ basic medium supplemented with 1.0 mg l^−1^ of 2,4-D and 0.5–10 mg l^−1^ of 6-BA was used for callogensis (Table 3). After callogenesis, callus was transferred to fresh medium every 10–15 days. All treatments were performed in triplicate, and each treatment consisted of 50 plantlets.

### FCM analysis

Nuclear suspensions of samples for flow cytometry (FCM) were prepared according to a previously reported method with minor modification (Doležel et al. 2007). The nuclear DNA content was determined using approximately 0.5 g leaves of mother plant, or 4-week-old callus. The raw materials for FCM analysis were finely chopped in 1 mL Otto I buffer in a petri dish at 4 °C. Final volume of each sample was adjusted to 500 μl after centrifugation. The ploidy level was determined by DAPI staining on a Partec CYFlow space. The staining solution, 500 μl DAPI-RNase solution (50 mg DAPI l^−1^ + 2 mg RNase l^−1^), was added to each sample. The mother-plant *T. chinensis* Rehd. var. *mairei* was used for the size standard of the ploidy level. All treatments were performed in triplicate.

### DNA extraction and PCR amplification

According to the manufacturer’s instructions, the genomic DNA of endosperm callus and leaves of mother plants were extracted and purified using a Plant DNA extraction reagent (TRIzol Reagent, Invitrogen). *Taxus cuspidata* var. nana LEAFY protein gene (GenBank accession No.: HQ245861) and *T. chinensis* 13-alpha-hydroxylase gene (GenBank accession No.: AY959321) were amplified from genomic DNA, and two pairs of primers were designed for PCR: LE2/f 5′-TGTGGCA AGTTTCTGCTTGA-3′, LE2/r 5′-TGCTTTGCATGCCTAAATACC-3′, t6/f 5′-TCAACATTCCCGGA TTCAGT-3′, t6/r 5′-TCCTTCCAAGCAATTTCGTC-3′, respectively. PCR was performed in a total volume of 20 μl, and the reaction mix consisted of each of the primers at a concentration of 0.5 μM, 10–50 ng of DNA template, and 10 μl 2 X Taq Plantinum PCR MasterMix (Tiangen, Beijing, China). The amplification scheme consisted of 35 cycles of 94 °C for 30 s, 61 °C for 30 s, and 72 °C for 45 s, and the final extension is 72 °C for 7 min. The amplification products were analyzed via 1.2 % agarose electrophoresis and visualized by ethidium bromide staining under UV.

### Sequencing and analysis

Half of the PCR products of endosperm callus and its mother plant were sequenced by Beijing Genomics Institution, in Beijing, China. And the other of them were ligated using by T1pEASY-T1 cloning kit (Tiangen, Beijing, China), in accordance with the manufacturer’s instructions. Transformed three mL of colony culture fluid, and the plasmid was sequenced by Beijing Genomics Institution, in Beijing, China. The DNA sequences were compiled using Chromas software. The online Clustal Omega program (http://www.ebi.ac.uk/Tools/msa/clustalo/) was applied to multiple sequence alignments.

### Data analysis

Data from callus induction, proliferation of endosperm callus and FCM was assessed by one-way analysis of variance (ANOVA), followed by Duncan’s POST-HOC test. A two-tailed P value of less than 0.05 was considered to be statistically significant. All analyses were conducted using SPSS 13.0.

## Results and discussion

### Hydropriming and light condition for callus induction

**Fig. 1:**
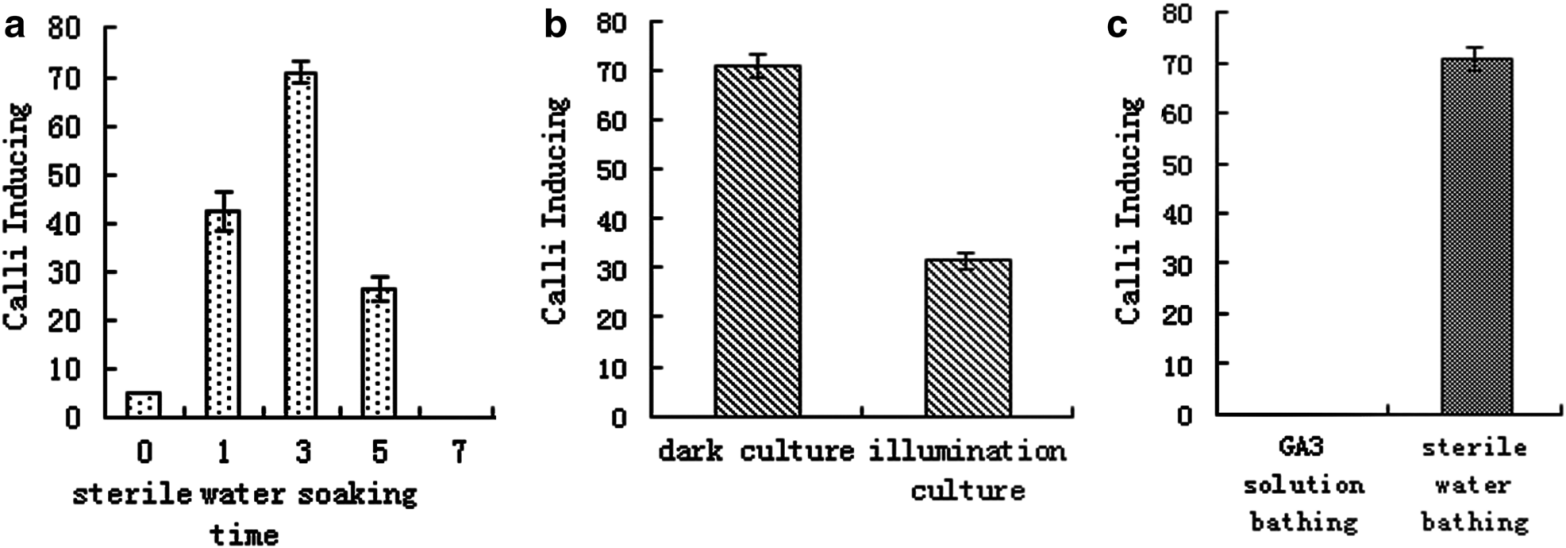
Effects of seed pre-treatments on callogenesis of *T. chinensis* Rehd. var. *mairei* after 4 weeks of *in vitro* culture. **a** Total ratio of callus induction with seed soaking in autoclaved deionized water for different time; **b** total ratio of callus induction culture under dark and illumination conditions; **c** total ratio of callus induction with seed soaking in autoclaved deionized water or 3 mg l^−1^ GA_3_ solution for 3 days

Inhibitors, including of abscisic acid, heptanoic acid, nonanoic acid and acetic acid in endosperm of *Taxus* genus, are strongly retardant the germination of seed (Le Page-Degivry 1973; Zhang et al. 2007). The hydropriming with water, which allows efficient dormancy broken on embryos have been reported (Zarek 2007; Zhiri et al. 1994). However, these inhibitors could be removed by hydropriming with sterile distilled water for endosperm callus induction, and optimal soaking time was 3 days (Fig. 1a). Compared with sterile distilled water, the GA_3_ solution couldn’t promote the inducible callus (Fig. 1c). Moreover, the inducible ratio was lower under light condition than in darkness (Fig. 1b).

### The efficiency of composition of medium on callus induction

Selection of suitable basic medium, and supplement of proper sort and concentration of plant growth regulators have shown to be the decisive factors on endosperm in vitro cultures (Thomas and Chaturvedi 2008). For the current study, the callus induction and browning ratio were obtained differently based on six dissimilar basic media supplemented with plant growth regulators at the same level in the endosperm of *T. chinensis* var. *mairei* (Table 1). The lowest callogenesis ratio of endosperm (8.68 %) was obtained from the SH basic medium, from which the lowest browning ratio was also reliable. Meanwhile, the higher callogenesis was achieved from media of B_5_ and WPM, and the callus induction ratio was 50.35 and 51.74 %, respectively. The B_5_ medium, with a lower browning ratio, and was followed by BLG, 1/2MS and MS.

**Table 1:** Effective of basic medium on callogenesis of *T. chinensis* Rehd. var. *mairei*

Basic medium	Callus inducing (%)	Browning ration (%)
BLG	45.83 ± 1.04^b^	21.10 ± 1.36^b^
B5	50.35 ± 1.59^a^	7.95 ± 0.25^e^
MS	39.93 ± 2.62^c^	25.01 ± 0.91^a^
WPM	51.74 ± 1.59^a^	16.74 ± 0.66^c^
SH	8.68 ± 0.60^d^	0.00 ± 0.00^f^
1/2MS	45.14 ± 2.17^b^	11.09 ± 0.55^d^

Means values within a column with the same letters are not significantly different at 5 % level by Duncan’s Mutiple Range Test

**Fig. 2:**
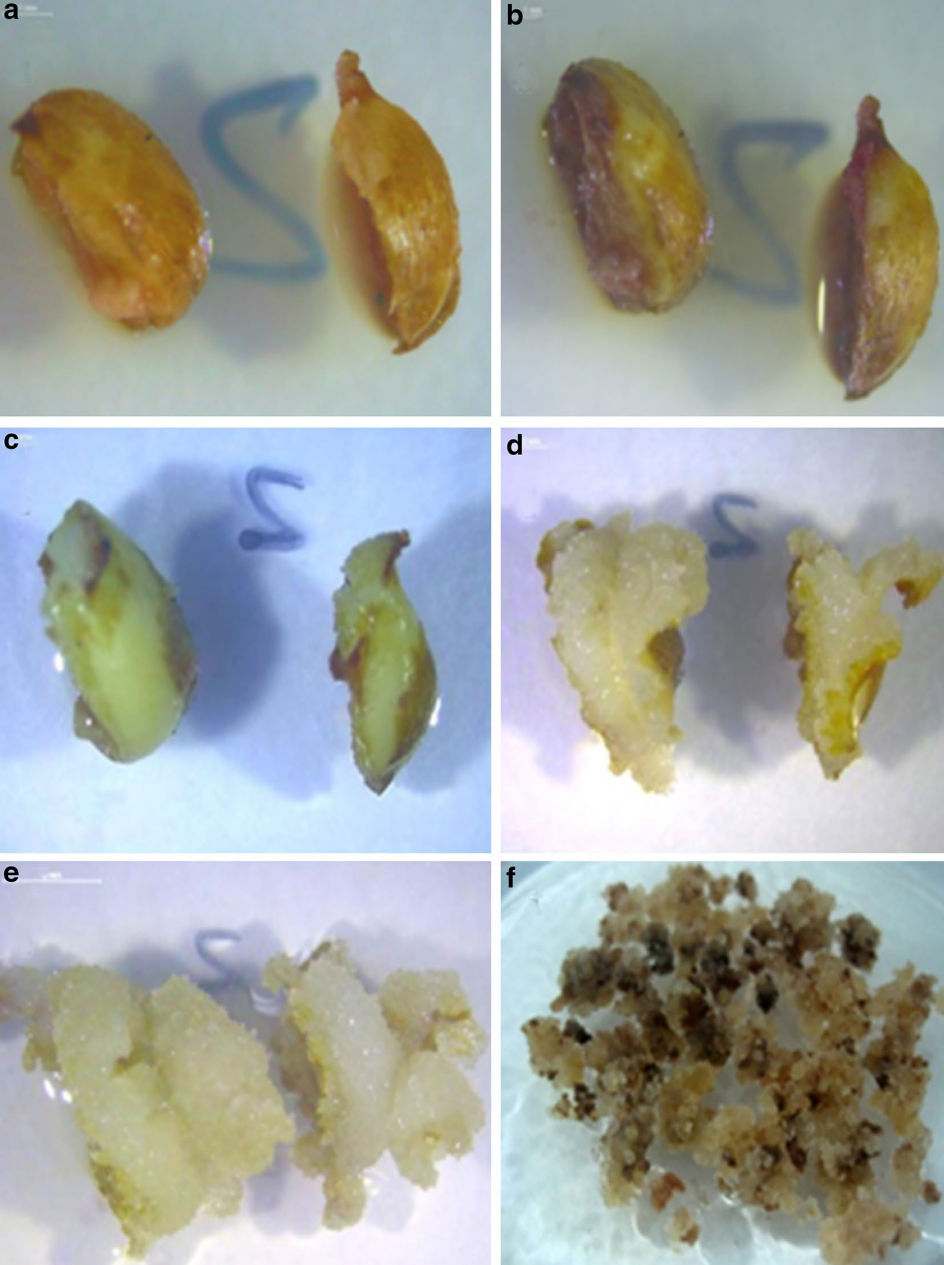
Aspects of *T. chinensis* Rehd. var. mairei callus induction from mature endosperm. **a** Endosperm callus induction on B_5_ medium for 5 days (*bar* 1 mm). **b** Endosperm callus induction on B_5_ medium for 10 days (*bar* 1 mm). **c** Endosperm callus induction on B_5_ medium for 18 days (*bar* 1 mm). **d** Endosperm callus induction on B_5_ medium for 23 days (*bar* 2 mm). **e** Endosperm callus induction on B_5_ medium for 30 days (*bar* 2 mm). **f** Endosperm callus subculture on the proliferation culture medium

The auxins concentration and sort had different effects on endosperm callus induction (Table 2). The control treatment without auxins and cytokinins was acquired a higher callus induction, but endosperm callus died during the subculture. In the meantime, the best callus induction of endosperm from B_5_ medium, supplemented with 2.5 mg 2,4-D l^−1^ and 0.5 mg 6-BA l^−1^ after 4 weeks of incubation, was observed with the value of 12.75-times higher in contrast with the lowest callus induction (Fig. 2a–f). Interestingly, the callus induction was reduced at a higher concentration of 2,4-D, and the same situation was observed in medium supplement with NAA (Table 2).

**Table 2:** Effective of auxins on endosperm callogenesis of *T. chinensis* Rehd. var. *mairei*

Medium (auxin concentration, mg l^−1^)	Ratio of induction (%)
B5	57.20 ± 2.65^b^
B5 + 2,4-D 1.0 + 6-BA 0.5	5.56 ± 2.41^f^
B5 + 2,4-D 2.5 + 6-BA 0.5	70.89 ± 2.31^a^
B5 + 2,4-D 3.5 + 6-BA 0.5	55.56 ± 2.41^bc^
B5 + 2,4-D 4.5 + 6-BA 0.5	30.83 ± 2.20^d^
B5 + NAA 1.0 + 6-BA 0.5	52.67 ± 2.32^c^
B5 + NAA 2.0 + 6-BA 0.5	27.03 ± 2.09^e^
B5 + NAA 4.0 + 6-BA 0.5	4.17 ± 0^g^

Means values within a column with the same letters are not significantly different at 5 % level by Duncan’s Mutiple Range Test

The callus induction was negatively correlated with the 6-BA concentration (Table 3). The highest callus induction was obtained in the treatment with 0.5 mg l^−1^ 6-BA and 2.5 mg l^−1^ 2,4-D. Moreover, no callogenesis was observed when 6-BA was supplemented at the highest concentration.

**Table 3:** Effective of cytokinin on callogenesis of *T. chinensis* Rehd. var. *mairei*

Medium (cytokinins concentration in mg l^−1^)	Ratio of induction (%)
B5 + 2,4-D 2.5 + 6-BA 0.5	70.89 ± 2.31^a^
B5 + 2,4-D 2.5 + 6-BA 1.0	47.22 ± 2.41^b^
B5 + 2,4-D 2.5 + 6-BA 2.0	30.56 ± 2.41^c^
B5 + 2,4-D 2.5 + 6-BA 4.0	15.28 ± 2.41_d_
B5 + 2,4-D 2.5 + 6-BA 6.0	9.72 ± 2.41^e^
B5 + 2,4-D 2.5 + 6-BA 10.0	0 ± 0^f^

Means values within a column with the same letters are not significantly different at 5 % level by Duncan’s Mutiple Range Test

### The ploidy analysis and homozygous identification of endosperm callus

**Fig. 3:**
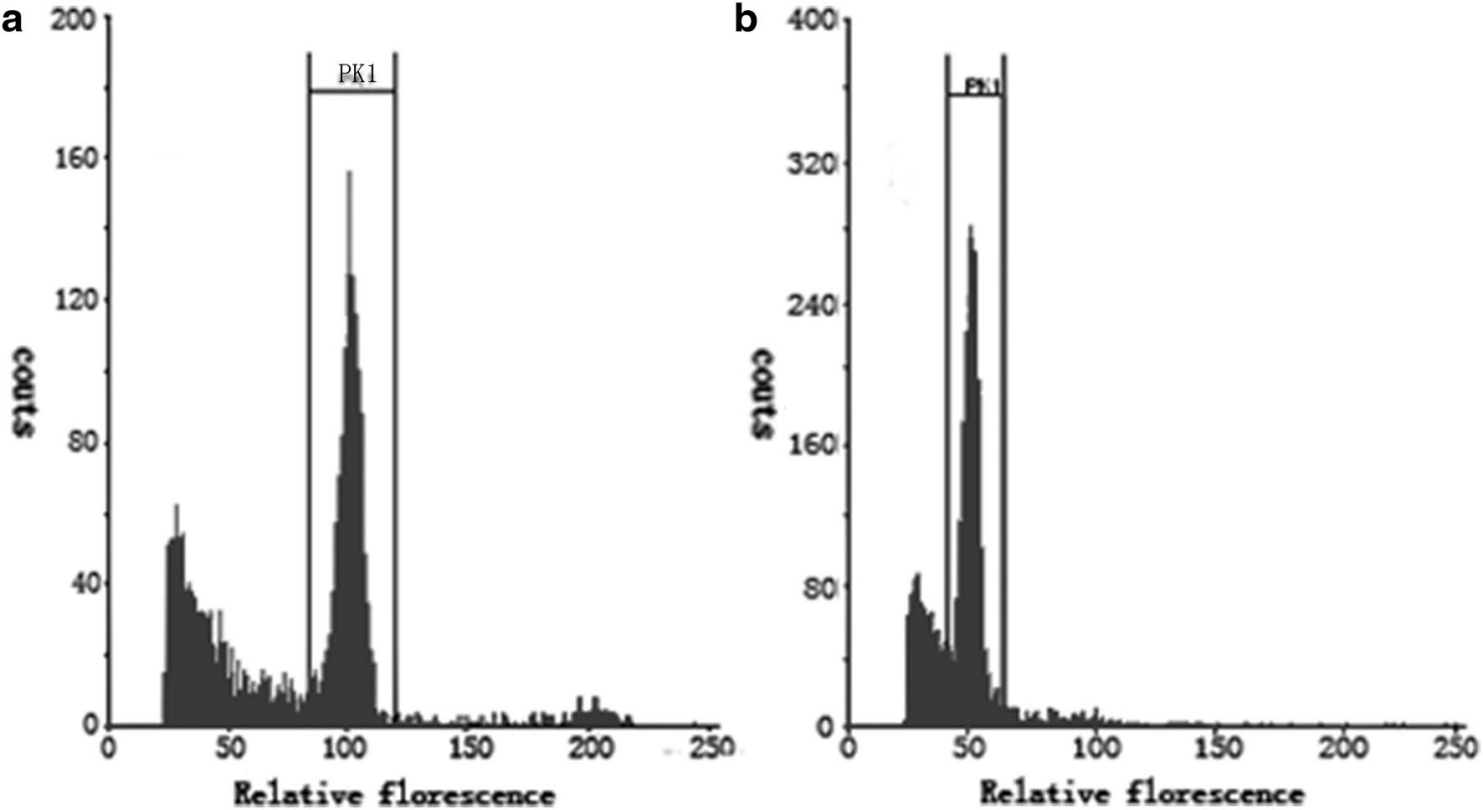
Histograms of nuclear DNA content obtained after FCM analysis of the DAPI-stained nuclei simultaneously isolated from the leaves and callus of endosperm of *T. chinensis* Rehd. var. *mairei.* **a** Leaves; **b** regenerated endosperm callus

The chromosome numbers of *Taxus* species is 24, and the haploid number is 12(Darlington and Dark 1932). The ploidy level can be assessed by chromosome counting and FCM analysis (Germanà 2011). FCM analysis is much easier, convenient and simplified on the determination of ploidy level (Gu et al. 2014; Carloni et al. 2014; Escobedo-GraciaMedrano et al. 2014). The nuclear DNA content, which has the same chromosome number measured by FCM in one genus, is correlated with chromosome numbers. In additional, the nuclear DNA content of genus in *Taxus* species are range from 22.3 to 24.3 pg (Zonneveld 2012), and the nuclear DAN content of the mother plant was in this range (the data was not shown in this paper). According to those, the ploidy level of endosperm callus and mother plant were investigated by FCM (Fig. 3a, b), and the results indicated that the callus was haploid callus.

**Fig. 4:**
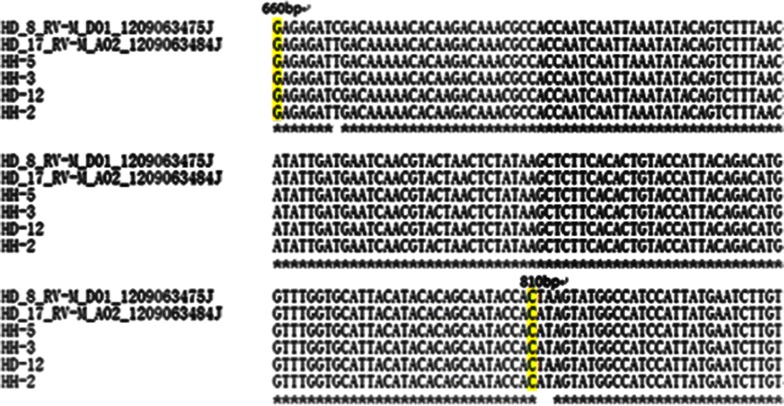
SNP detection from direct sequencing of amplification PCR and PCR plasmid of colonies from endosperm of callus and mother plants by primers of LE2 (*T. cuspidata* var. nana LEAFY protein gene, GenBank accession No.: HQ245861). Three SNPs were detective by aligning with CLUSTAL OMEGA. The HD-12 is sequence of diploid PCR amplification, and two sequences are diploid PCR amplification of plasmid of colonies, including of HD_8_RV-M_D01_12090643475J and HD_17_RV-M_A02_12090643484J. The HH-2, HH-3 and HH-5 are the sequences of haploid PCR amplifications

**Fig. 5:**
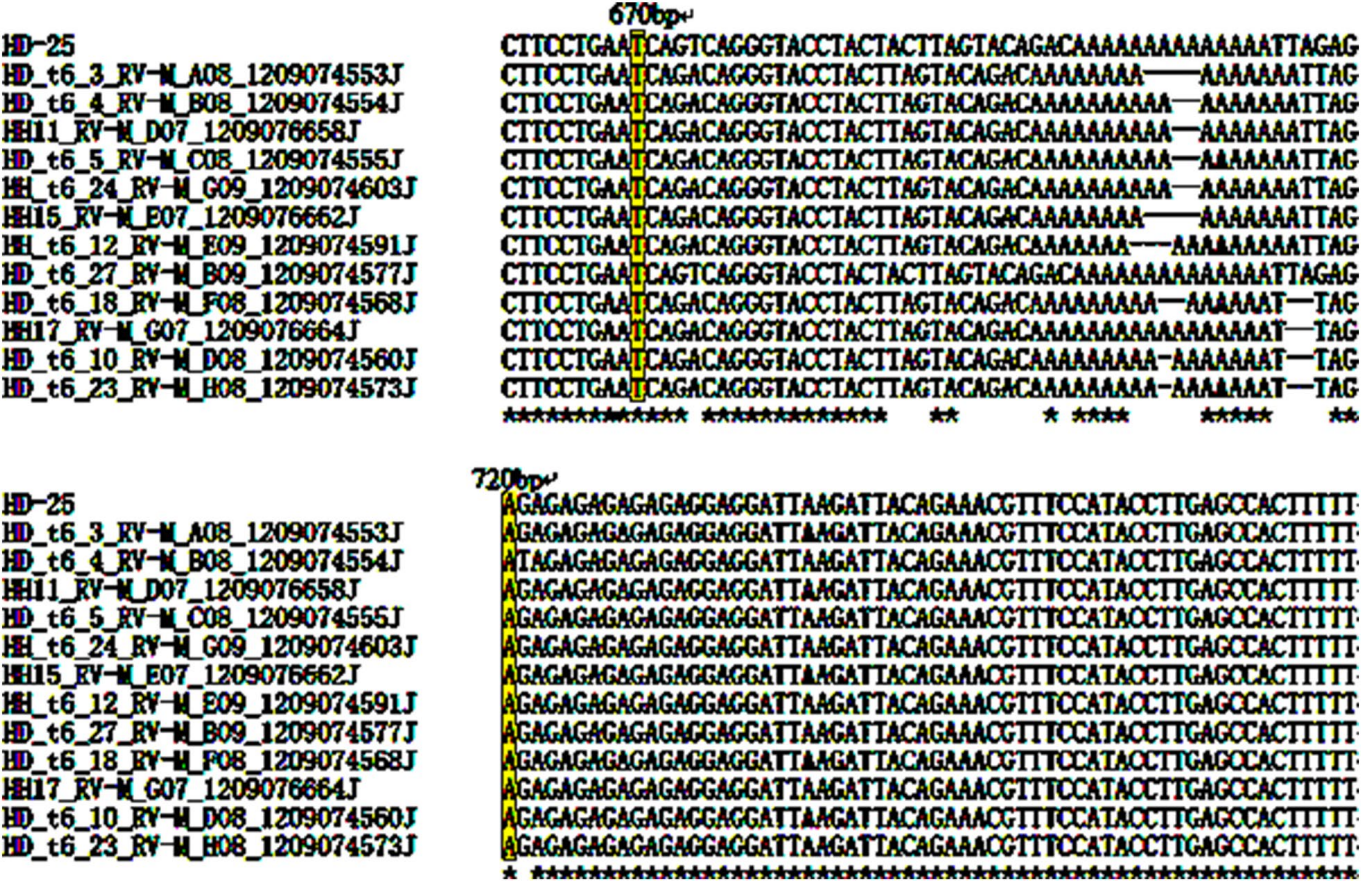
SNP detection from direct sequencing of PCR amplification and PCR plasmid of colonies from endosperm of callus and mother plants by primers of t6 (*T. chinensis* 13-alpha-hydroxylase gene, GenBank accession No. AY959321). Sixteen SNPs were detective by aligning with CLUSTAL OMEGA. The HD-25 is sequence of diploid PCR amplification, and seven sequences are diploid PCR amplification of plasmid of colonies, including of HD_t6_3_RV-M_A08_1209074553J, HD_t6_4_R V-M_B08_1209074554J, HD_t6_5_RV-M_C08_1209074555J, HD_t6_27_RV-M_B09_1209074577J, HD_t6_18_RV-M_F08_1209074568J, HD_t6_10_RV-M_D08_1209074560J, HD_t6_23_R V-M_H08_1209074573J. Five sequences are haploid PCR amplification of plasmid of colonies, including of HH_ 11_RV-M_D07_1209076658J, HH15_RV-M_E07_1209076662J, HH12_RV-M_G 07_1209076664J, HH_t6_12_RV-M_E07_1209 074591J, HH_t6_24_RV-M_G07_1209074603J

Isozyme analyses, random amplified polymorphic DNA (RAPD) markers and microsatellites have been utilized to assess to homozygosis of regenerated callus and plants (Rai and Shekhawat 2014). The single nucleotide polymorphism (SNP) markers is accelerated application along with the deployment of next-generation sequencing (NGS)(Thakur et al. 2014). But some projects don’t have entire genomic information, and focus on limited number of SNPs in candidate gene studies. Under this circumstances, the very inexpensive, highly flexible SNP genotyping methods, including of homogenous allele-specific PCR-based SNP genotyping method in candidate gene, are needed (Wang et al. 2005). Li et al. (2015) obtained 103 SNPs from 9 pairs of amplification PCR productions, and 84 of them are detected to be homozygous in the 26 loquat (*Eriobotrya japonica*) cultivars. Our studies were designed to evaluate the LEAFY protein gene and 13-alpha-hydroxylase gene polymorphisms between diploid mother-plant and haploid endosperm of callus. Homozygotic and heterozygotic loci were detected in different PCR amplification production or plasmid of colonies after the sequencing quality was verified by Chromas software (Figs. 4, 5). Three SNPs were detective among three PCR amplification productions in endosperm callus, and one PCR amplification production and two plasmid colonies of diploid plant localizing LEAFY protein gene, including of three transversions in T/C (1) and A/T (2) (Fig. 4). 16 SNPs were examined among five plasmid colonies of endosperm callus, one PCR amplification production and seven plasmid colonies of diploid plant localizing 13-alpha-hydroxylase gene, consisting of 15 transversions in A/T (6), A/C (4), A/G (2), G/T (3), and one transiton in (G/C) (Fig. 5). The results indicated that the genome typing of endosperm callus was homozygous, and the diploid plant was heterozygous with LEAFY protein gene and 13-alpha-hydroxylase gene of SNP loci analysis.

## Conclusion

We have provided a protocol of homozygous haploid of callus induction. The endosperm of callus was obtained by combination with hydropriming, optimal media, and concentrations of plant growth regular. The results of FCM and PCR-based SNP analysis were provided as an important foundation for further studies on genomic, taxiod-metabolism of functional genomic and bio-fermentation engineering of *Taxus* plants and other species in secondary metabolisms.
